# Anlotinib added to third generation EGFR tyrosine kinase inhibitor therapy for advanced NSCLC patients with oligo-progression: a retrospective study (ALTER-L058)

**DOI:** 10.3389/fphar.2025.1686364

**Published:** 2025-11-07

**Authors:** Fei Zhou, Minglei Zhuo, Hongmin Wang, Nong Yang, Jisheng Li, Shi Jin, Zhengxiang Han, Guilin Zeng, Jun Liu, Yang Song, Kangwu Wang, Dabing Huang, Ling Li, Jian Chen, Jinghui Bai, Fengming Ran, Caicun Zhou

**Affiliations:** 1 Department of Oncology, Shanghai East Hospital, Tongji University School of Medicine, Shanghai, China; 2 Department of Thoracic Oncology, Beijing Cancer Hospital, Beijing, China; 3 Department of Respiratory Medicine, The First Affiliated Hospital of Zhengzhou University, Zhengzhou, China; 4 Department of Oncology, The Second People’s Hospital of Hunan, Changsha, China; 5 Department of Medical Oncology,Cancer Center, Qilu Hospital of Shandong University, Jinan, China; 6 Department of Oncology, Cancer Hospital & Shenzhen Hospital, Chinese Academy of Medical Sciences, Shenzhen, China; 7 Department of Oncology, The Affiliated Hospital of Xuzhou Medical University, Xuzhou, China; 8 Department of Oncology, Chengdu Fifth People’s Hospital, Chengdu, China; 9 Department of Respiratory and Critical Care Medicine, Guangzhou First People’s Hospital, Guangzhou, China; 10 Department of Oncology, Tangdu Hospital, Fourth Military Medical University, Xi’an, China; 11 Department of Thoracic Surgery, The First Affiliated Hospital of Bengbu Medical University, Bengbu, China; 12 Department of Oncology, The First Affiliated Hospital of USTC, Division of Life Sciences and Medicine, University of Science and Technology of China, Hefei, China; 13 Department of Oncology, Tengzhou Central People’s Hospital, Tengzhou, Shandong, China; 14 Department of Oncology, The Affiliated Yantai Yuhuangding Hospital of Qingdao University, Yantai, China; 15 Medical Oncologist, Liaoning Cancer Hospital, Shenyang, China; 16 Department of Thoracic Oncology, Hubei Cancer Hospital, Tongji Medical College, Wuhan, China

**Keywords:** non-small cell lung cancer, oligoprogression, epidermal growth factor receptor tyrosine-kinase inhibitor, antiangiogenic therapy, anlotinib

## Abstract

**Objective:**

To investigate the efficacy of anlotinib, an antiangiogenic multikinase inhibitor, as an add-on therapy to first-line epidermal growth factor receptor (EGFR) tyrosine-kinase inhibitor (TKI) for patients with *EGFR*-mutant non-small cell lung cancer (NSCLC) who were previously untreated before first-line EGFR TKI but subsequently developed oligoprogression.

**Methods:**

This multicenter, retrospective cohort study (ALTER-L058) analyzed data from the electronic health records-derived de-identified systems at 16 cancer centers in China. Adult patients between 18 and 75 years of age with histologically or cytologically confirmed locally advanced or metastatic NSCLC who received first-line third-generation EGFR TKI monotherapy and had an oligoprogressive disease were included. Eligible patients received anlotinib (8, 10 or 12 mg) on days 1–14 of each 3-week cycle for ≥6 cycles. Tumor response was assessed radiologically by investigators per RECIST, version 1.1. The primary outcome was investigators-assessed progression-free survival, calculated from the date of medication initiation for the oligoprogressive disease to the first documented progressive disease or death.

**Results:**

Between January 2020 and December 2023, 100 patients received EGFR TKI plus anlotinib and 50 received EGFR TKI. At the data cutoff (20 November 2024), the median progression-free survival was 9.23 months (95% CI, 8.94–10.87) with EGFR TKI plus anlotinib *versus* 5.42 months (95% CI, 4.83–6.80) with EGFR TKI (hazard ratio [HR] = 0.38, 95% CI, 0.26–0.56; log rank test, *P* < 0.0001), meeting the primary endpoint. Anlotinib was generally well tolerated, with manageable adverse events.

**Conclusion:**

Anlotinib, when added onto EGFR TKI therapy following gradual progression or oligo-progression, conferred significant PFS benefits upon *EGFR* mutant NSCLC patients, supporting adding anlotinib to ongoing first-line EGFR TKI therapy for oligoprogressive disease.

## 1 Introduction

Lung cancer is the most prevalent cancer, accounting for one in eight cancer cases worldwide, and remains a principal cause of cancer death in both sexes globally (Bray et al., 2024). China is experiencing an increasing burden of lung cancer cases, with approximately 1,060,600 new cases and 733,300 deaths in 2022, accounting for 21.98% of new cancer cases and 28.49% of cancer deaths in China (Han et al., 2024). Activating epidermal growth factor receptor (*EGFR*) mutations exon 19 deletions (ex19del) and exon 21 L858R mutations make up 85%–90% of non-small cell lung cancer (NSCLC) cases (Gazdar, 2009). Targeting actionable oncogenic driver alterations, which occur in approximately 60% of lung cancer cases, remains a cornerstone in targeted therapy for NSCLC (Hanna et al., 2020).

The advent of EGFR tyrosine-kinase inhibitors (TKIs) is paradigm changing for the treatment of *EGFR* mutant NSCLC patients. Currently, third-generation EGFR TKIs, which are marked for their potent central nervous system (CNS) penetrance, are the standard-of-care (SOC) first-line treatment for advanced-stage *EGFR*-mutant NSCLC (Cheng et al., 2024; Soria et al., 2018; Ramalingam et al., 2020; Chen et al., 2022). In China, apart from osimertinib, furmonertinib, a pan-EGFR TKI with CNS antitumor activity, and aumolertinib, formerly almonertinib, an oral EGFR-TKI that is selective for mutant EGFR over wild-type EGFR, have demonstrated efficacy for *EGFR* mutated NSCLC and were approved in China for the treatment of locally advanced, metastatic NSCLC with confirmed EGFR T790M mutation whose disease has progressed during or after EGFR TKI therapy (Shi et al., 2022; Bao et al., 2016; Lu et al., 2022; Yang et al., 2020). However, in untreated *EGFR*-mutant NSCLC patients, third-generation EGFR TKIs only lead to a modest gain in overall survival (OS) compared to first-generation EGFR TKIs. In addition, the median progression-free survival (PFS) of *EGFR*-mutant NSCLC patients is less than 2 years (Ramalingam et al., 2020) with first-line third-generation EGFR TKIs and disease progression eventually ensues. Two major patterns of progression after initial response to systemic therapy have been reported (systemic progression and oligoprogression): in contrast to widespread metastases involving multiple sites (polymetastatic disease), patients may exhibit different kinetics of disease progression and have a controlled primary tumor and 1 to 5 metachronous metastases (Hellman and Weichselbaum, 1995; Niibe and Hayakawa, 2010; Niibe and Chang, 2012; Xu et al., 2021). A retrospective analysis of 148 *EGFR* mutant advanced NSCLC patients across 13 Swiss centers showed that 77% experienced oligoprogressive disease following treatment with first line osimertinib, a third-generation EGFR TKI, and they had a longer OS than patients who had systemic progressive disease (Schuler et al., 2024). In the multicenter, real-world FLOWER Study in Italy, 44 of 126 *EGFR* mutant advanced NSCLC patients had disease progression and 20.1% of them had oligoprogression and 54.5% had systemic progression (Lorenzi et al., 2022). In the multicenter, real-world Reiwa Study in Japan, 344 (59.0%) of 583 *EGFR* mutant advanced NSCLC patients had disease progression and 20.1% of them had oligoprogression and 54.5% patients who received first-line osimertinib developed progressive disease and 156 (45.4%) of them had oligoprogressive disease (Watanabe et al., 2024).

As oligoprogressive disease is characterized by localized progression of a maximum of three to five metastatic lesions while the primary disease and lesions in other areas remain under control or stable (Patel et al., 2019), its treatment goals and treatment strategies are different from systemic progressive disease. Local ablative radiotherapy with or without systemic therapy, continuation of systemic therapy or combination therapy may be pursued depending on the oligometastatic disease state and the treatment goals (Guckenberger et al., 2020; Iyengar et al., 2023; Lim, 2021). In the Reiwa Study, approximately half (49.5% [77/156]) of the patients with oligoprogressive disease continued osimertinib therapy. Emergence of resistance to third-generation EGFR TKI is one of the major causes of treatment failure and resistance mechanisms involve EGFR-dependent and independent pathways (Cooper et al., 2022). The vascular endothelial growth factor (VEGF)-VEGF receptor (VEGFR) signaling pathway plays a key role in driving oncoangiogenesis in lung cancer. Dual blockade of EGFR and VEGF-VEGFR signaling represents a promising therapeutic strategy in the treatment of EGFR mutant NSCLC patients with oligoprogressive disease given extensive crosstalk between these pathways in oncoangiogenesis and drug resistance (Zhou et al., 2025; Choi et al., 2022). Several retrospective studies have explored bevacizumab for EGFR mutant NSCLC patients who had oligoprogressive disease after first-line EGFR TKI therapy, but with rather limited data on third-generation EGFR TKI(Long et al., 2022; Yu et al., 2021; Xu et al., 2022).

Unlike bevacizumab that only targets the VEGFR signaling pathway, anlotinib is an oral multikinase inhibitor (MKI) that simultaneously suppresses VEGFR, fibroblast growth factor receptor (FGFR), platelet-derived growth factor receptor (PDGFR), and c-Kit (Shen et al., 2018; Lin et al., 2018). Currently, it is approved in China as third and later-line treatment for advanced NSCLC based on the results of the ALTER 0303 trial (Han et al., 2018). An exploratory subgroup analysis of this trial showed that anlotinib significantly improved PFS and OS of patients with both sensitive *EGFR* mutations and wild-type EGFR (Zhao et al., 2022). The clinical activities of anlotinib in *EGFR* mutant NSCLC patients with oligoprogressive disease following first or second-line EGFR TKI therapy were explored in the single arm phase 2 ALTER-L001 trial showing that the addition of anlotinib to EGFR TKI therapy led to a median PFS of 9.1 months and an overall response rate (ORR) of 6.7% (Chen et al., 2025). Meta-analytic evidence indicates that combining anlotinib with docetaxel improves response and PFS in advanced NSCLC, supporting the feasibility of multi-agent anlotinib-based regimens (Hetta et al., 2025c). Numerous trials also showed effective anlotinib-containing combination regimens, such as anlotinib combined with PD-1 blockades (Chu et al., 2025; Shi et al., 2025), anlotinib plus third generation EGFR TKI (Wang et al., 2025) have shown promising efficacy in terms of improved survival outcomes. Recent pharmacological reviews further elucidate the multitarget and microenvironment-modulating actions of anlotinib that can potentiate EGFR-TKI efficacy (Hetta et al., 2025b). In a retrospective study of 121 *EGFR* mutant NSCLC patients with gradual progression following first-line EGFR TKI, the addition of anlotinib extended the median PFS by 3 months over EGFR TKI alone (6.7 vs. 3.6 months, *P* < 0.001) (Xiang et al., 2024).

Oligo-progression is seldomly evaluated as a study endpoint in clinical trials. To augment insight from the ALTER-L001 trial and to increase evidence on the choice of anlotinib as an add-on therapy to ongoing systemic therapy in the context of primary treatment, we carried out this real-world study to investigate the efficacy and safety of anlotinib for the treatment of *EGFR* mutant NSCLC patients who developed gradual or oligo-progression while on first-line third-generation EGFR TKI therapy. This is the first real-world, multicenter Chinese cohort specifically evaluating anlotinib with third-generation EGFR-TKIs in oligoprogressive NSCLC.

## 2 Materials and methods

### 2.1 Ethics

The study was approved by the ethics committee of Shanghai East Hospital, the lead institution (2024YS-252). No patient consent was required given the nature of the retrospective study. Patient data were anonymized in this report.

### 2.2 Cohort selection

This retrospective, real-world cohort study utilized data from the electronic health records-derived de-identified systems at 16 cancer centers in China. De-identified patient-level structured data such as demographics and unstructured data including disease characteristics, treatment, treatment response and survival outcomes were curated via technology-enabled chart abstraction. Adult patients between 18 and 75 years of age with histologically or cytologically confirmed locally advanced or metastatic NSCLC who received first-line third-generation EGFR TKI monotherapy between January 2020 and December 2023 (≤2 cycles of intercalating chemotherapy were allowed) and had a gradual progression or oligoprogressive disease were included. Gradual progression was defined as a controlled primary tumor for ≥6 months with EGFR TKI monotherapy, no target lesion progression (non-target lesion progression and/or newly developed metastases), and no deterioration of clinical symptoms (Chen et al., 2025). Oligo-progression was defined as a controlled primary tumor for ≥3 months with EGFR TKI monotherapy, progression limited to ≤5 sites (solitary extracranial lesion or intracranial lesion confined within the radiation field), and no deterioration of clinical symptoms. For the convenience of this report, gradual progression and oligoprogression are referred to as oligoprogressive disease. Other eligibility criteria were evidence of an Eastern Cooperative Oncology Group (ECOG) performance status score of 0–1, at least one measurable lesion per Response Evaluation Criteria in Solid Tumors (RECIST) version 1.1 within the preceding 3 months, *EGFR* mutations, including *EGFR* 19del and exon 21 L858R (exon 20ins excluded), based on local testing or reports from a non-participating hospital.

Patients were ineligible if they had primary resistance to third-generation EGFR TKIs (<3 months of treatment) or if they had dramatic progression, defined as a controlled primary tumor for ≥3 months with EGFR TKI monotherapy, rapid deterioration in clinical symptoms, and marked target lesion progression in comparison with the preceding assessment (Chen et al., 2025), during first-line third-generation EGFR TKI monotherapy. Other exclusion criteria were the presence of druggable targets such as *MET* amplification and EGFR C797X mutation, and small cell lung cancer (including mixed small cell lung cancer and NSCLC).

Patients with a controlled primary tumor for ≥3 months with a third-generation EGFR TKI who had a gradual progression or oligoprogressive disease continued to receive the EGFR TKI at the same dose and schedule at the discretion of the investigators. In addition, eligible patients should have received oral anlotinib (8, 10 or 12 mg; Chia-tai Tianqing Pharmaceutical Co., Ltd.) on days 1–14 of each 3-week cycle for ≥6 cycles and have radiological data. Patients who received concurrent or sequential systemic therapy including chemotherapy or antiangiogenic therapy with bevacizumab were excluded.

### 2.3 Assessments and outcomes

Tumor response was assessed radiologically by investigators per RECIST, version 1.1; PFS was calculated from the date of medication initiation for the primary disease (PFS2) or the oligoprogressive disease (PFS) to the first documented progressive disease or death, whichever occurred earlier. The primary outcome of the study was investigators-assessed PFS. The secondary outcomes included ORR, defined as the proportion of patients who had a complete or partial response as their best overall response to anlotinib, disease control rate, defined as the proportion of patients who had a complete or partial response or a stable disease, and OS, defined as the time from the date of medication initiation for gradual or oligo-progression to the date of death of any cause.

Adverse events (AEs) were monitored using the National Cancer Institute Common Terminology Criteria for Adverse Events version 5.0 (NCI CTCAE 5). The occurrences, frequencies, and severities of treatment-related (TRAEs) were tabulated, and all AEs were described using the latest version of MedDRA preferred terms and CTCAE grade.

### 2.4 Statistical analysis

Based on literature, the median PFS was 3.1(Park et al., 2016), 5.4 (Soria et al., 2015) and 5.4 months (Soria et al., 2015), respectively, for patients who were treated with single agent erlotinib, an EGFR TKI plus platinum-containing doublet regimen, or standard platinum-containing doublet regimen after emergence of resistance to EGFR TKIs. In the ALTER-L001 trial, the median PFS was 9.2 months for patients with gradual, local or oligo-progression who were treated with anlotinib with continued EGFR TKI therapy (Chen et al., 2025). Based on previous studies and the ALTER-L001 trial (Soria et al., 2015; Park et al., 2016; Chen et al., 2025), a target sample size of 150 was anticipated that would provide 82% power with a one-sided α of 0.05, corresponding to a median PFS of 9.2 months and 5.4 months for the EGFR TKI plus anlotinib group (100 patients) and the EGFR TKI group (50 patients), respectively.

Efficacy was based on the full analysis set (FAS) that included all patients who met the study eligibility criteria. PFS and other time-to-event end points were analyzed using the Kaplan-Meier method and the corresponding 95% CIs were calculated and compared using log-rank test. The ORR and disease control rate, along with their two-sided 95% CIs, were estimated for each group using the Clopper-Pearson method and compared by Chi-square test or Fisher exact test.

The safety set included all patients who met the study eligibility criteria and had postbaseline safety data. AEs were summarized using descriptive statistics.

Statistical analysis was done using SAS 9.4 (The SAS Institute, Cary, NC).

## 3 Results

### 3.1 Patient characteristics

**FIGURE 1:**
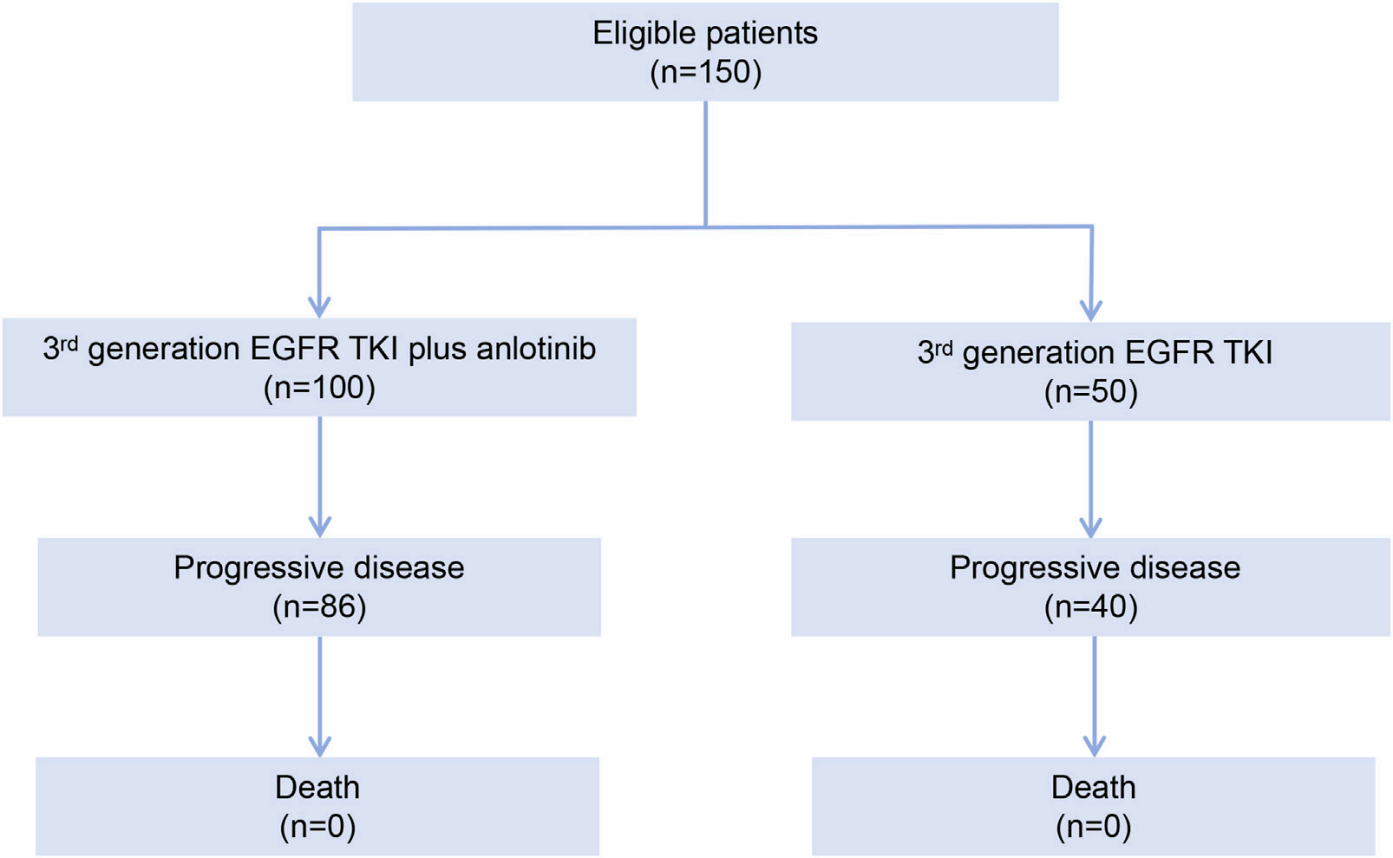
Patient disposition flowchart.

Between January 2020 and December 2023, 712 patients were screened for eligibility and 150 patients were eligible for the study, including 100 in the EGFR TKI plus anlotinib group and 50 in the EGFR TKI group (Figure 1). The patients had a median age of 59 years and 38.0% were male. Most patients (95.3%) had clinical stage IV disease. Forty-four (29.3%) patients had brain metastasis. Ninety-five (63.3%) patients in the overall population had *EGFR* ex19del mutation and 55 (36.7%) harbored *EGFR* ex21L858R mutation. Prior third generation EGFR TKI therapy included osimertinib (76.0% [114/150]), aumolertinib (14.7% [22/150]) and furmonertinib (9.3% [14/150]). The demographic and baseline characteristics were well balanced between the two groups (Table 1).

**TABLE 1:** Patient baseline characteristics.

Characteristic	EGFR TKI plus anlotinib (n = 100)	EGFR TKI (n = 50)	*P* ^a^
Age, median (IQR), years	59 (53–64)	62 (52–67)	*P* = 0.163
Sex			
Male	38 (38.0)	19 (38.0)	*P* = 1.000
Female	62 (62.0)	31 (62.0)	
Pathologic types			
Adenocarcinoma	99 (99.0)	50 (100.0)	*P >* 0.999
Adenosquamous carcinoma	1 (1.0)	0 (0)	
Smoking status			
Never smokers	70 (70.0)	36 (72.0)	*P =* 0.895
Former smokers	27 (27.0)	12 (24.0)	
Current smokers	3 (3.0)	2 (4.0)	
ECOG performance status score			
0	23 (23.0)	11 (22.0)	*P =* 0.888
1	77 (77.0)	39 (78.0)	
*EGFR* mutational status			
Exon 19 deletion	63 (63.0)	32 (64.0)	*P =* 0.920
L858R	37 (37.0)	18 (36.0)	
Tumor staging			
IIIB	5 (5.0)	2 (4.0)	*P* > 0.999
IV	95 (95.0)	48 (96.0)	
CNS metastases			
Yes	29 (29.0)	15 (30.0)	*P =* 0.888
No	71 (71.0)	35 (70.0)	
Third-generation EGFR TKI			
Osimertinib	77 (77.0)	37 (74.0)	*P =* 0.956
Aumolertinib	14 (14.0)	8 (16.0)	
Furmonertinib	9 (9.0)	5 (10.0)	

Data are expressed as number (%) unless otherwise indicated.

^a^ Two independent sample t-test for continuous variables; Fisher exact test for categorical variables.

Definitions:

Eastern Cooperative Oncology Group (ECOG) performance-status scores range from 0 to 5, with higher numbers indicating increasing impairment in activities of daily living.

Never smokers, defined as smoking <100 cigarettes/lifetime; former smokers, defined as abstinence from smoking for at least 15 years on the day before the start of therapy; current smokers, defined as smoking >100 cigarettes/lifetime, or smoking >100 cigarettes/lifetime but abstinence from smoking for less than 1 year on the day before the start of therapy for oligoprogressive disease.

### 3.2 Efficacy

**FIGURE 2:**
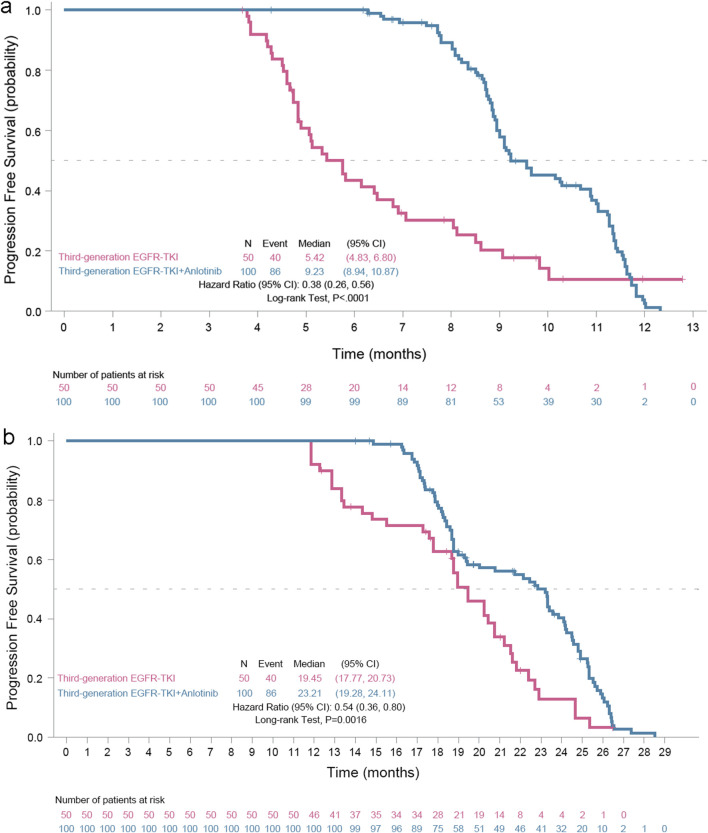
Kaplan-Meier progression-free survival (PFS) curves of EGFR mutant advanced nonsmall cell cancer patients with oligoprogressive disease. **(a)** PFS, the primary study outcome, was calculated from the date of medication initiation for the oligoprogressive disease and **(b)** PFS2, was calculated from the date of medication initiation for the primary disease to the first documented progressive disease or death, whichever occurred earlier.

A total of 86 and 40 investigators-confirmed PFS events occurred from the date of medication initiation for the oligoprogressive disease to the data cutoff date (20 November 2024) in the EGFR TKI plus anlotinib group and the EGFR TKI group, respectively. The median PFS was 9.23 months (95% CI, 8.94–10.87 months) in the EGFR TKI plus anlotinib group *versus* 5.42 months (95% CI, 4.83–6.80 months) in the EGFR TKI group, meeting the primary study endpoint (Figure 2a). The addition of anlotinib to EGFR TKI led to a 62% reduction in the risk of progression (hazard ratio [HR] = 0.38, 95% CI 0.26–0.56; log rank test, *P* < 0.0001).

Meanwhile, 86 and 40 investigators-confirmed PFS events occurred from the date of medication initiation for the primary disease to the data cutoff date in the EGFR TKI plus anlotinib group and the EGFR TKI group, respectively. The median PFS2 was 23.21 months (95% CI 19.28–24.11 months) for the EGFR TKI plus anlotinib group vs. 19.45 months (95% CI 17.77–20.73 months) for the EGFR TKI group, with a 46% reduction in the risk of progression (HR 0.54, 95% CI 0.36–0.80, *P* = 0.0016) (Figure 2b).

In the FAS, no patients in either group had a complete response. Seven patients in the EGFR TKI plus anlotinib group showed a partial response while none in the EGFR TKI group had an objective response. The investigators-assessed ORR was 7.0% (95% CI 3.4%–13.8%) for the EGFR TKI plus anlotinib group and 0% for the EGFR TKI group. In addition, 84 patients in the EGFR TKI plus anlotinib group had a stable disease and the disease control rate was 91.0% (95% CI 83.8%–95.2%). Seventeen patients in the EGFR TKI group had a stable disease and the disease control rate was 34.0% (95% CI 54.2%–79.2%).

OS data was still immature. No deaths were reported in either group.

### 3.3 Safety

Treatment-related AEs (TRAEs) of any grade occurred in 92 patients (92.0%) in the EGFR TKI plus anlotinib group and 46 patients (92.0%) in the EGFR TKI group, including grade ≥3 TRAEs in 37 patients (37.0%) in the EGFR TKI plus anlotinib group and 16 patients (34.0%) in the EGFR TKI group (Table 2). The most frequently reported grade 3 or worse TRAEs included hypertension (12.0%), γ-glutamyl transferase increased (5.0%) and hyponatremia (5.0%) in the EGFR TKI plus anlotinib group and dry skin, paronychia and appetite decreased, each occurring in 8.0% patients in the EGFR TKI group (Table 2).

**TABLE 2:** Treatment-related adverse events occurring in at least 10% of the patients in either group in the safety analysis population.

	EGFR TKI plus anlotinib (n = 100)		EGFR TKI (n = 50)	
Preferred terms	Any grade	Grade 3 or worse	Any grade	Grade 3 or worse
Hypertension	68 (68.0)	12 (12.0)	0	0
Diarrhea	50 (50.0)	1 (1.0)	24 (48.0)	2 (4.0)
Rash	50 (50.0)	1 (1.0)	25 (50.0)	2 (4.0)
Hypertriglyceridemia	45 (45.0)	2 (2.0)	0	0
Thyroid stimulating hormone increased	43 (43.0)	1 (1.0)	0	0
Palmar–plantar erythrodysaesthesia syndrome	43 (43.0)	3 (3.0)	0	0
Hypercholesterinemia	42 (42.0)	1 (1.0)	0	0
Cough	35 (35.0)	1 (1.0)	8 (16.0)	2 (4.0)
Fatigue	30 (30.0)	1 (1.0)	6 (12.0)	2 (4.0)
Dry skin	30 (30.0)	0	16 (32.0)	4 (8.0)
Paronychia	30 (30.0)	0	15 (30.0)	4 (8.0)
Proteinuria	28 (28.0)	2 (2.0)	0	0
Oral mucositis	25 (25.0)	0	13 (26.0)	1 (2.0)
Appetite decreased	25 (25.0)	0	10 (20.0)	4 (8.0)
γ-glutamyl transferase increased	29 (29.0)	5 (5.0)	0	0
Hyperbilirubinemia	24 (24.0)	2 (2.0)	0	0
Hemoptysis	20 (20.0)	3 (3.0)	0	0
Low density lipoprotein increased	19 (19.0)	1 (1.0)	0	0
Hyponatremia	19 (19.0)	5 (5.0)	0	0
Oropharyngeal dysphagia	18 (18.0)	0	0	0
Pruritus	18 (18.0)	0	9 (18.0)	1 (2.0)
Constipation	15 (15.0)	0	7 (14.0)	1 (2.0)
Vomiting	15 (15.0)	0	7 (14.0)	1 (2.0)
Body weight decreased	15 (15.0)	0	0	0
Headache	13 (13.0)	0	6 (12.0)	1 (2.0)
Hematuria	12 (12.0)	0	0	0
Respiratory difficulty	12 (12.0)	0	6 (12.0)	0
Anemia	12 (12.0)	0	6 (12.0)	1 (2.0)
Vomiting	11 (11.0)	0	5 (10.0)	0
Fever	11 (11.0)	0	5 (10.0)	0
Urinary tract infection	11 (11.0)	0	0	0
Upper respiratory tract infection	11 (11.0)	0	5 (10.0)	0

Data are expressed as number (%).

Treatment-related adverse events are described using the latest version of MedDRA, preferred terms and National Cancer Institute Common Terminology Criteria for Adverse Events version 5.0 (NCI CTCAE, 5) grade.

Serious AEs occurred in 12 (12.0%) patients in the EGFR TKI plus anlotinib group and 6 patients (12.0%) in the EGFR TKI group. TRAEs led to dose reductions in 23 patients (23.0%) in the EGFR TKI plus anlotinib group and 4 (8.0%) in the EGFR TKI group. TRAEs led to treatment interruptions in 25 patients (25.0%) in the EGFR TKI plus anlotinib group and 10 (20.0%) in the EGFR TKI group.

## 4 Discussion

Oligoprogressive disease following EGFR TKI therapy in *EGFR* mutant NSCLC patients leads to different clinical outcomes from systemic progressive disease and also has different treatment goals. Less than 30% of the patients in the ALTER-L001 trial received third-generation EGFR TKI (Chen et al., 2025). In this real-world study, all patients received first-line third-generation EGFR TKI systemic therapy. The addition of antiangiogenic agent anlotinib led to a 3.76-month extension of median PFS from medication initiation in patients with oligoprogressive disease compared to continued EGFR TKI monotherapy. Anlotinib was well tolerated and did not lead to a notable increase in the incidences of grade 3 or worse TRAEs and serious AEs. We felt that gradual or oligo-progression could offer a narrow therapeutic window that allows physicians to intervene early to change the failure treatment patterns from oligoprogressive disease to systemic progressive disease. Identifying *EGFR* mutant NSCLC patients with oligoprogressive disease and providing maintenance therapy with anlotinib would lead to significant PFS gains.

In the randomised phase 3 FLAURA trial involving previously untreated patients with *EGFR* mutant NSCLC, osimertinib attained a median PFS of 18.9 months (Soria et al., 2018), which is comparable to the median PFS (19.5 months (95% CI 18.0–20.9 months)) of our patients who received EGFR TKI only. Seventy-six percent of our patients received first-line osimertinib. The 3.8-month extension in the median PFS EGFR TKI plus anlotinib overo EGFR TKI only occurred in *EGFR* mutant NSCLC patients who have already developed resistance while on first-line third-generation EGFR TKI, a class known for deep and durable responses. Extending PFS by 3.8 months in a post-resistance setting could be viewed in the context of NSCLC for which PFS gains of 2–4 months have historically supported regulatory approvals (e.g., osimertinib vs*.* erlotinib/gefitinib in FLAURA showed a 5.5-month PFS gain (Soria et al., 2018)). The 62% reduction in the risk of progression (HR = 0.38, 95% CI 0.26–0.56) is substantially greater than the 46% reduction following the start of treatment of the primary disease, suggesting that anlotinib may be particularly effective in targeting angiogenic escape mechanisms in oligoprogressive. By adding anlotinib rather than switching therapies, clinicians can maintain EGFR TKI exposure, which continues to suppress sensitive clones. This strategy delays clonal evolution and systemic progression, a key goal in precision oncology, while targeting clones that escape EGFR TKI through angiogenic escape mechanisms. The greater efficacy of EGFR TKI plus anlotinib post-oligoprogression suggests that angiogenesis may be a dominant resistance pathway in the subset of patients with gradual or oligo-progression, validating VEGFR/PDGFR inhibition with anlotinib as a rational combination strategy. However, real-world heterogeneity might influence the outcomes.

In the ALTER-L001 trial, patients who received third-generation EGFR TKI and anlotinib maintenance had a median PFS of 9.2 months (95% CI 6.7–12.6 months) (Chen et al., 2025). This is in line with the median PFS (9.23 months, 95% CI, 8.94–10.87 months) of patients in the EGFR TKI plus anlotinib group observed in the current real-world study. Most patients (90.8%) in the ALTER-L001 trial had gradual progression and 18.0% had brain metastasis. This real-world study has a similar prolife of patients. The favorable outcomes among patients with brain metastases in our cohort are consistent with recent meta-analyses demonstrating benefit of anlotinib–radiotherapy combinations in NSCLC with intracranial disease (Hetta et al., 2025a). In the retrospective study by Xiang *et al.*, the median PFS of *EGFR* mutant NSCLC patients with gradual progression following first-line EGFR TKI who received anlotinib was 6.7 months compared to 3.6 months for those on continued EGFR TKI alone (Xiang et al., 2024). Their study only enrolled patients with gradual progression (disease control for ≥6 months) while our study included both patients with gradual progression and oligoprogression (disease control ≥3 months). Patients with disease control for ≥6 months are more likely to have metachronous oligometastatic disease, which is associated with a less aggressive disease phenotype and a better prognosis than synchronous oligometastatic disease (Ashworth et al., 2014). Currently, there is not a universally accepted definition for oligoprogressive disease. We limited progression to ≤5 sites, which is in line with most studies that use an arbitrary cutoff of 3–5 progressive lesions (Guckenberger et al., 2020).

Overall, the addition of anlotinib to EGFR TKI therapy did not notably increase the incidence of toxicities, with comparable rates of grade 3 or worse TRAEs and serious AEs between the two groups. The safety profile of anlotinib plus EGFR TKI is similar to previous report (Zhou et al., 2024). No new safety concerns emerged during the study.

The study has several limitations. This study was based on retrospective cohorts with potential bias in patient selection and non-randomized comparison, and the study findings need to be prospectively validated in randomized trials. There is also lack of molecular profiling (e.g., EGFR T790M mutation status, EGFR C797X) of the patients. Regarding OS, the number of events was immature; OS results will be reported upon data maturity. Osimertinib was shown to be superior to first-generation EGFR TKIs in OS (38.6 months vs. 31.8 months) (Ramalingam et al., 2020). Given accumulating trial evidence and our increased understanding of the multitarget and microenvironment-modulating actions of anlotinib (Chu et al., 2025; Shi et al., 2025; Wang et al., 2025; Hetta et al., 2025b) and the ever important role of biomarkers in guiding cancer therapy, we anticipate that future studies will address the important issues of the efficacy and safety of anlotinib combined with PD-1 blockades or other targeted therapy in the treatment of NSCLC patients with oligoprogressive disease, with elucidation of and guidance by predictive biomarkers. In conclusion, real-world evidence is important in identifying and addressing the gap between trials and real-world practice. The current real-world study shed insight into the effectiveness and safety of anlotinib as an add-on therapy to ongoing systemic therapy in the context of primary treatment in *EGFR* mutant NSCLC patients who developed gradual or oligo-progression while on first-line third-generation EGFR TKI therapy. The study demonstrated that anlotinib, when added onto EGFR TKI therapy following gradual progression or oligo-progression, conferred significant PFS benefits upon *EGFR* mutant NSCLC patients, adding to the growing evidence on the choice of anlotinib as an effective treatment after gradual progression or oligoprogression while patients are receiving first-line EGFR TKI therapy. The findings of this study will be very relevant and guide physicians for informed treatment of patients with *EGFR* mutant NSCLC, though prospective randomized validation of the study findings is required. Our findings support further exploration of combination regimens guided by molecular and imaging biomarkers.

## Data Availability

The original contributions presented in the study are included in the article/supplementary material, further inquiries can be directed to the corresponding author.
